# Stability of Curcumin on Amphiphilic Chitosan

**DOI:** 10.1021/acsomega.5c01178

**Published:** 2025-05-23

**Authors:** Alessandra L. Poli, Brenda G. Fanchiotti, Juliana S. Gabriel, Anderson M. Arandas, Carla C. Schmitt

**Affiliations:** a Instituto de Química de São Carlos, 153988 Universidade de São Paulo, Caixa Postal 780, São Carlos, SP 13560-970, Brazil; b Instituto Federal de Educação, Ciência e Tecnologia do Amapá − IFAP, Rodovia BR 210, Km 103, Zona Rural, Porto Grande, AP 68997-000, Brasil

## Abstract

The stability of curcumin (Cur) in different media, such as buffer, modified chitosan, and solvents, was evaluated by using UV–vis and fluorescence. An amphiphilic derivative of chitosan (modified chitosan) was synthesized by substituting diethylamino and dodecyl groups. The critical aggregation concentration of modified chitosan (ChM) was determined by using pyrene as a hydrophobic probe. The ChM aggregates at concentrations above 1.1 × 10^–2^ g·L^–1^, and hydrophobic environments are formed. For Cur in the presence of ChM (Cur/ChM), it can be seen that the keto form has spectral characteristics, indicating that ChM is stabilizing the Cur molecules in the keto form. These results agree with Cur absorbance spectra obtained in different solvents, in which the tautomeric equilibrium of Cur is shifted to the keto form in nonpolar solvents. The fluorescence intensity of Cur in ChM (at concentrations above the CAC) increased significantly, unlike that of Cur in the buffer. The quantum yield value obtained for Cur was 0.05 g·L^–1^ of ChM was higher than that of Cur in the other media. At concentrations of ChM above 0.011 g·L^–1^, it aggregates and can incorporate the Cur molecule into the hydrophobic portion, promoting stabilization. This behavior can be confirmed by fluorescence micrographs, which reveal the fluorescent domains of curcumin in the hydrophobic environments of ChM. The percentages of Cur degradation were 96% in the buffer and 71% in 0.05 g·L^–1^ Ch, 93% in 0.005 g·L^–1^ ChM, 60% in 0.01 g·L^–1^ ChM, and 30% in 0.05 g·L^–1^ ChM. It is possible to notice that the Cur degradation percentage is 0.05 g·L^–1^ ChM (above CAC concentration) was lower compared with the degradation of Cur in the other media. Therefore, these results indicate that the ChM above the CAC, in its aggregated form, can protect the dye molecule from the effects of water.

## Graphical abstract

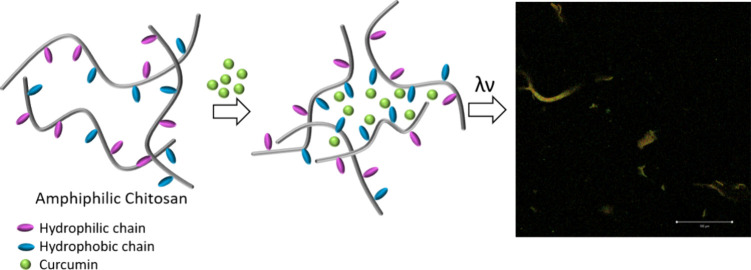

## 1 Introduction

Curcumin (Cur) is a polyphenolic compound found in turmeric, which exists in keto–enol equilibrium (Figure 1),^1^ and has been widely studied for its numerous benefits to health, including anti-inflammatory, antioxidant, and anticancer properties.^2–4^ In neutral and acidic solutions, Cur is found in its keto form.^1^ On the other hand, in alkaline media, the enol form is predominant.

**Figure 1:**
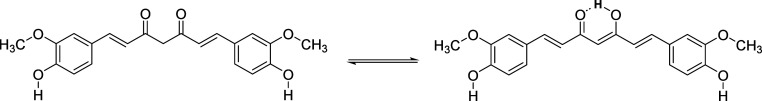
Keto–enol equilibrium of curcumin.

However, its poor solubility in water and low stability in acidic and alkaline environments limit its therapeutic potential.^5,6^ On the other hand, Cur is more soluble and presents better results for photophysical processes in nonpolar environments.^7^

To enhance the bioavailability of Cur, various delivery systems have been developed,^8–10^ including the use of amphiphilic chitosan nanoparticles.^11–14^

Chitosan (Ch) is a natural biopolymer derived from chitin, which is the second most abundant biopolymer in nature. It has enhanced biocompatibility, biodegradability, mucoadhesion, and low toxicity, making it an attractive material for drug delivery.^15^ However, its hydrophilic nature limits its use as a carrier for hydrophobic drugs like Cur.

To overcome this challenge, Ch can be modified to create amphiphilic Ch, which has both hydrophilic and hydrophobic properties, making it suitable for loading and delivering hydrophobic drugs.^16,17^

Amphiphilic Ch can self-assemble in aqueous solutions, forming nanoparticles with a hydrophilic outer shell and a hydrophobic inner core.^18^ Cur can be encapsulated within the hydrophobic core of amphiphilic Ch nanoparticles, enhancing its solubility and bioavailability.^12^

Amphiphilic Ch microemulsion was efficient for Cur loading and transdermal delivery, which is demonstrated by extremely high drug loading, improved drug stability, and excellent transdermal efficiency.^19^

Many works have studied the release of Cur from Ch,^17,18,20,21^ but little attention has been paid to the stability of Cur, which is of fundamental importance for its various applications. This work focuses on evaluating the spectroscopic properties of Cur incorporated into modified Ch and its stability as a function of time in the face to degradation process.

In this way, the stability of curcumin in buffer, commercial chitosan, and amphiphilic derivative synthesized from depolymerized chitosan was followed using spectroscopic techniques.

## 2 Materials and Methods

### 2.1 Materials

Cur and Ch (viscosity-average molar mass 93,000; deacetylation degree 85%) used in this work were obtained from Aldrich. Glacial acetic acid and sodium acetate were purchased from Synth.

### 2.2 Amphiphilic Chitosan Preparation

The amphiphilic Ch derivative with 49 and 17% substitution by diethylaminoethyl (DEAE) and dodecyl (DD) groups, respectively, was prepared as previously reported by Gabriel et al.^22^ (Figure 2). ^1^HNMR spectroscopy (data not shown) was used to determine substitution degrees, and the results were Ch containing 49% DEAE groups and 17% DD groups.

**Figure 2:**
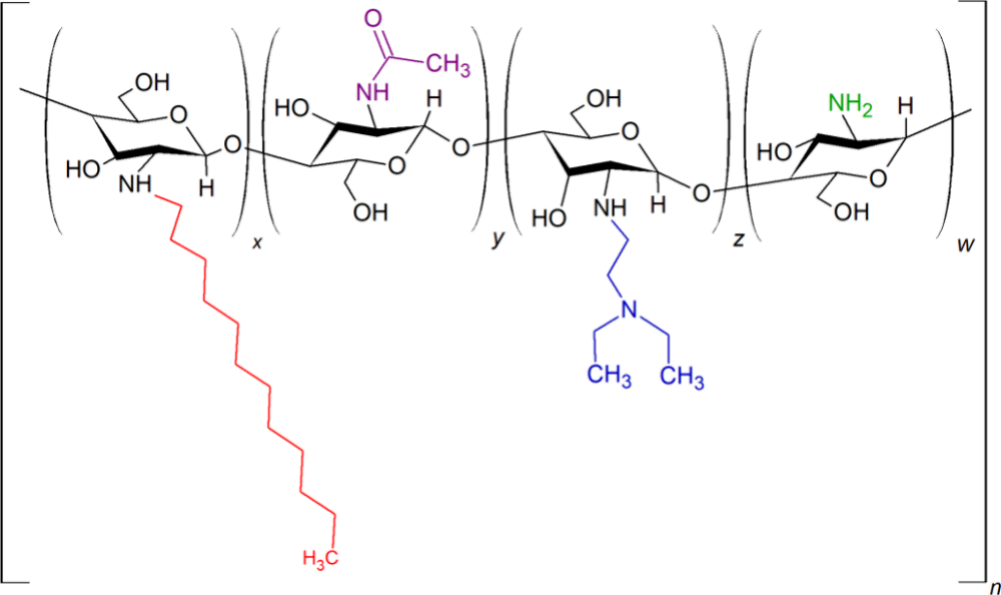
Structural representation of amphiphilic chitosan. *x* represents the content of DEAE substituted, *y* represents the remaining acetylated, *z* represents the N­(C_2_H_2_)­(C_2_H_5_)_2_ substituted, and *w* represents the remaining deacetylated fractions.

### 2.3 Determination of Critical Aggregation Concentration (CAC)

The CAC of this amphiphilic Ch was determined in the same way as in previous works,^23^ through fluorescence measurements using pyrene as a hydrophobic fluorescence probe.

### 2.4 Spectroscopy Measurements

A stock solution of Cur was prepared in ethanol. Then, this solution was diluted to 2.5 × 10^–5^ mol L^–1^ in different media, such as acid acetic 0.3 mol·L^–1^/sodium acetate 0.2 mol·L^–1^ buffer at pH 4.5, commercial Ch, amphiphilic Ch, and organic solvents.

Suspensions in different concentrations were prepared by dispersing commercial and amphiphilic chitosans in 0.3 mol·L^–1^ acetic acid/0.2 mol·L^–1^ sodium acetate buffer at pH 4.5 under stirring.

UV–vis spectra were obtained on a Shimadzu UV-2550 spectrophotometer in the 300–600 nm range. Fluorescence measurements were made by using a Hitachi F-4500 spectrofluorimeter with excitation at 420 nm.

Fluorescence quantum yields were determined relative to riboflavin as a fluorescence standard (eq 1).^24^ Thus, the fluorescence emission spectrum of Cur in different media and that of the standard were used in the calculations, considering the known quantum yield of the standard (Φ^0^_F_ = 0.3).^25^

$$\Phi_{F}=\frac{\int I(\lambda )\partial \lambda }{\int I(\lambda {)}^{0}\partial \lambda }\times \frac{O.D.^{0}}{O.D.}\times \frac{n^{2}}{n^{0^{2}}}\times \Phi_{F}^{0}$$ 1

where Φ^0^_F_ = 0.3 is the fluorescence quantum yield of the riboflavin standard at 298 K; O.D.^0^ and O.D. are the optical densities of the standard and the sample at 420 nm; *I* and *I*_o_ are the corresponding fluorescence emission integrals, and *n* and *n°* are the refractive indexes. The samples and the reference solution were excited at λexc = 420 nm.

### 2.5 Confocal Fluorescence Images

Confocal fluorescence micrographs of Cur in acidic acetic solution: 0.3 mol·L^–1^/sodium acetate 0.2 mol·L^–1^ buffer and amphiphilic Ch were made using a Zeiss LSM 780 confocal microscope (Carl Zeiss, Jena, Germany) with numerical aperture NA = 0.8, 20×. A 488 nm argon laser was used as the excitation source.

### 2.6 DLS Measurements

Dynamic light scattering (DLS) of Cur/ChM acid acetic acid 0.3 mol·L^–1^/sodium acetate 0.2 mol·L^–1^ buffer was performed using a Malvern Zetasizer Nano ZS (Malvern Instruments).

## 3 Results and Discussion

### 3.1 CAC Determination of Amphiphilic Chitosan

The aggregation of the amphiphilic Ch derivative was followed by using pyrene as a fluorescent probe. The *I*_1_/*I*_3_ band ratio of the pyrene fluorescence emission spectrum is used to determine the CAC, as this ratio provides information about the polarity of the environment. Figure 3 shows a graph of the *I*_1_/*I*_3_ ratio of pyrene fluorescence bands as a function of the amphiphilic Ch concentration in the buffer.

**Figure 3:**
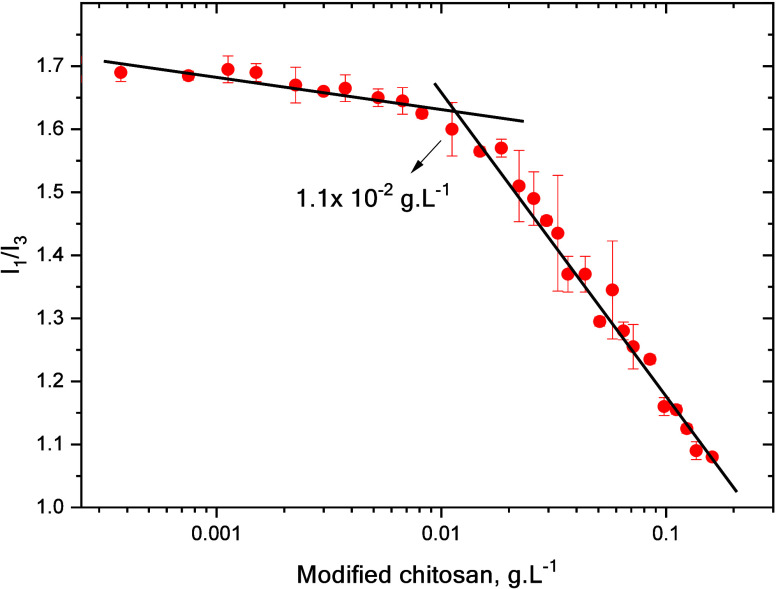
*I*_1_/*I*_3_ ratio of pyrene fluorescence bands as a function of amphiphilic chitosan concentration, 0.3 mol·L^–1^ acetic acid/0.2 mol. L^–1^ sodium acetate buffer at pH 4.5.

According to these results, amphiphilic Ch aggregates at concentrations above 1.1 × 10^–2^ g·L^–1^, and hydrophobic environments are formed.

### 3.2 Spectroscopic Behavior of Curcumin in Different Media

The absorption spectrum of Cur 0.3 mol·L^–1^ acetic acid/0.2 mol·L^–1^ sodium acetate buffer at pH 4.5 shows a broad band at 425 nm and a shoulder at 360 nm (Figure 4a). These bands correspond to π→π* electronic transitions.

**Figure 4:**
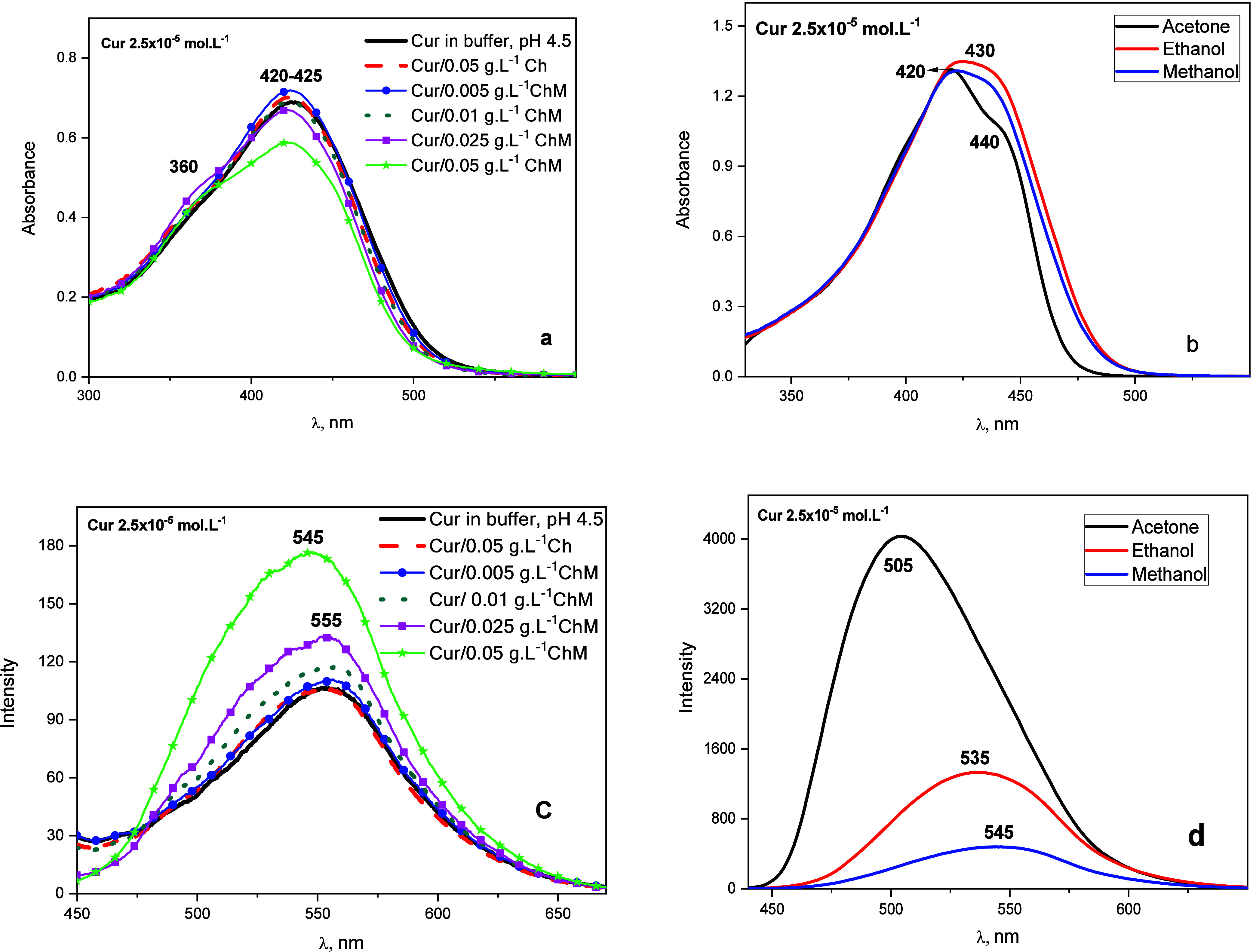
(a) UV–vis absorption and (c) and fluorescence emission spectra of curcumin in 0.3 mol·L^–1^ acid acetic/0.2 mol·L^–1^ sodium acetate buffer at pH 4.5, chitosan, and different concentrations of modified chitosan. (b) UV–vis absorption spectra of curcumin in solvents. (d) Fluorescence emission spectra of curcumin in solvents.

The band in the range from 420 to 425 nm is characteristic of the enolic form in solution.^26,27^ It was also possible to observe a shoulder at 360 nm related to the keto form.

A decrease in the intensity of the bands as the concentration of ChM increases, accompanied by a hypsochromic shift and the formation of a shoulder at 360 nm, could be observed in the absorption spectra of Cur in the presence of modified Ch (Cur/ChM). Thus, the blue shift, band formation at 360 nm (corresponding to the keto form), and absorbance intensity decrease at 420–425 nm indicate that ChM stabilizes the Cur molecules in the keto form. Furthermore, at concentrations of ChM above 0.011 g L^–1^, it is aggregated and can incorporate the Cur molecule in the hydrophobic portion.

These results agree with the Cur absorbance spectra obtained in different solvents, in which a decrease in the band at 430 nm was observed and a shift to blue was observed with a reduction in solvent polarity. The tautomeric equilibrium of Cur is shifted to the keto form in nonpolar solvents (Figure 4b).

The fluorescence emission spectra (Figure 4c) of Cur in buffer, Ch, and lower ChM concentrations showed a band at 555 nm. With increasing concentration of ChM (ChM = 0,05 g·L^–1^, above CAC), this band shifts to 545 nm.

The fluorescence intensity of Cur in modified Ch (in a concentration above CAC) increased significantly, unlike Cur in the presence of buffer. This indicates that Cur has a greater affinity to the hydrophobic sites formed in the aggregation of modified Ch. These results corroborate the fluorescence results obtained for Cur as a function of the solvent polarity. In solvents with a higher nonpolar character, the fluorescence spectrum of Cur exhibited a smaller Stokes shift (Figure 4d).

DD groups in ChM promote the formation of hydrophobic environments that can surround the chromophore molecules, providing greater stability.

The quantum yield value was obtained for Cur at 0.050 g·L^–1^ of ChM was higher than for Cur in the other media (Table 1). This concentration of ChM corresponds to its aggregated form (above the CAC), where more hydrophobic environments for Cur are present. Among the solvents used, the highest quantum yield value was for Cur in acetone, which agrees with the results previously reported in literature.^8^

**Table 1:** Fluorescence Quantum Yield Value (Φ_F_) for Cur in Different Media

medium	**Φ**_**F**_ **/10** ^**–4**^
buffer (pH 4.5)	6.0 ± 0.2
0.05 g·L^–1^ Ch	6.0 ± 0.3
0.01 g·L^–1^ ChM	13.0 ± 0.9
0.05 g·L^–1^ ChM	23.0 ± 2.0
ethanol	1000 ± 30
methanol	330 ± 12
acetone	3000 ± 89

This behavior can be confirmed by fluorescence micrographs presented in Figure 5. In the fluorescence images of Cur in buffer and at low concentrations of Ch (Figure 5a,b), it is possible to notice a uniform yellow-green emission due to the presence of Cur in the medium. At higher concentrations of Ch (Figure 5c–f), in which Ch is aggregated, the images reveal the fluorescent domains of Cur in the hydrophobic environments of the modified Ch.

**Figure 5:**
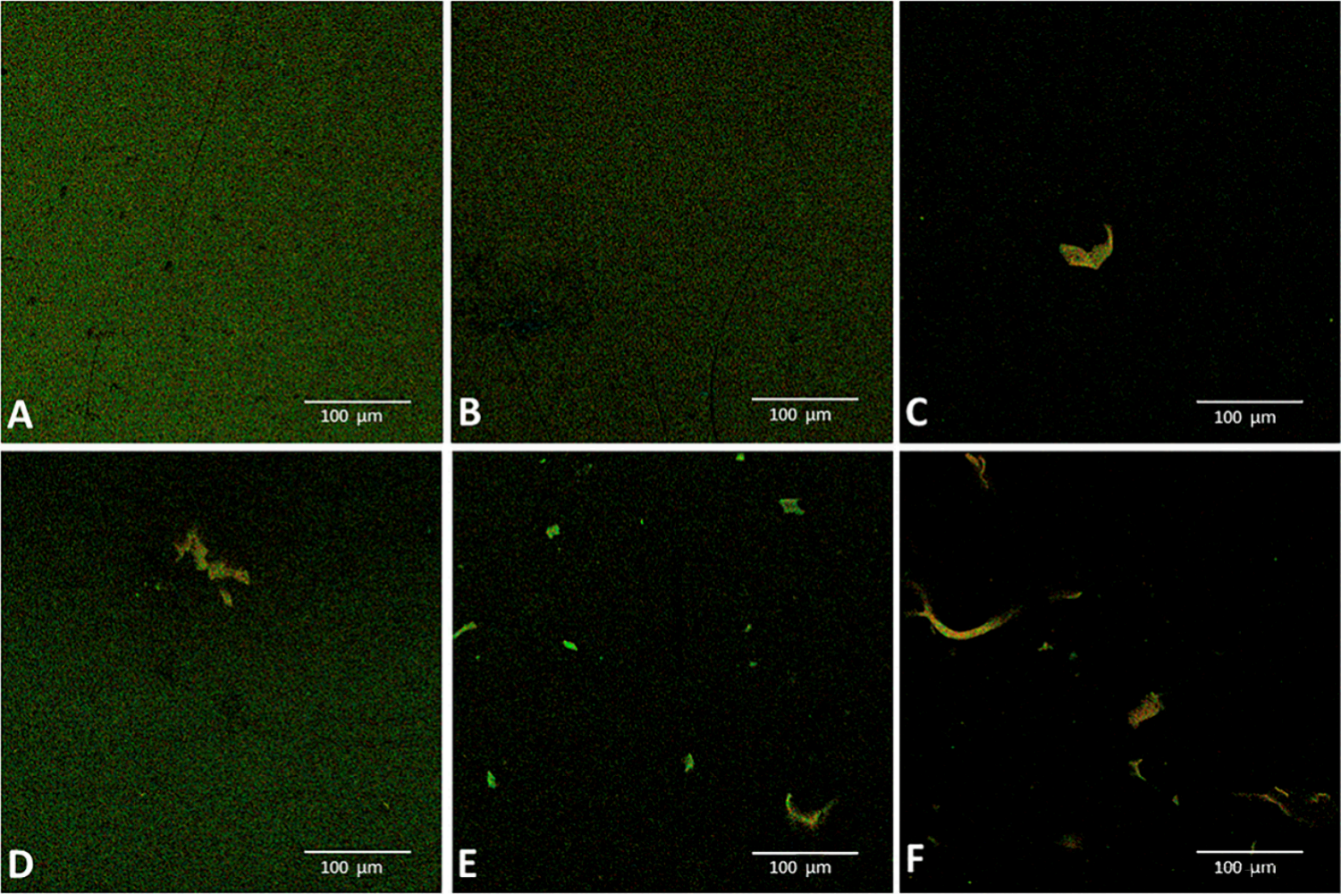
Confocal fluorescence images for (a) Cur/buffer, (b) Cur/0.005 g·L^–1^ ChM, (c) Cur/0.01 g·L^–1^ ChM, (d) Cur/0.01 g·L^–1^ ChM, (e) Cur/0.1 g·L^–1^ ChM, and (f) Cur/0.1 g·L^–1^ ChM. Excitation at 488 nm.

It is possible to observe red spots in the images. To explain that, it is necessary to remember that in solution, chitosan is a wet agglomerate with a mixture of acetylated and free amine containing glucopyranoside rings, in which basic microenvironments are formed.

In addition, the fluorescence emission spectrum for Cur in alkaline medium, NaOH solution at pH 9.0 (Figure 1S), shows a band between 500 and 700 nm, including the red region. The fluorescence emission spectrum was obtained with an excitation wavelength of 488 nm, the same excitation wavelength that was used to obtain the confocal images. Red spots in the agglomerates indicate that the modified Ch aggregates contain domains with a higher concentration of amine groups (basic environment) and that Cur, when associated with these amine-rich domains, exhibits red fluorescence, as shown in Figure 1S.

DLS was employed to assess the particle size of modified Ch at concentrations below, near, and above the CAC (Figure 6). Cur/ChM at a low concentration of ChM (Cur/0.005 g·L^–1^ChM) presents a unique particle size distribution with a modal diameter of 122 nm. With increasing modified Ch concentration (concentration at the start of aggregation), the particle size distribution becomes bimodal, with diameters at 51 and 220 nm. For the ChM concentration above its CAC, a new population is formed at a particle size of 1484 nm. The size distribution presents diameters at 51, 220, and 1484 nm.

**Figure 6:**
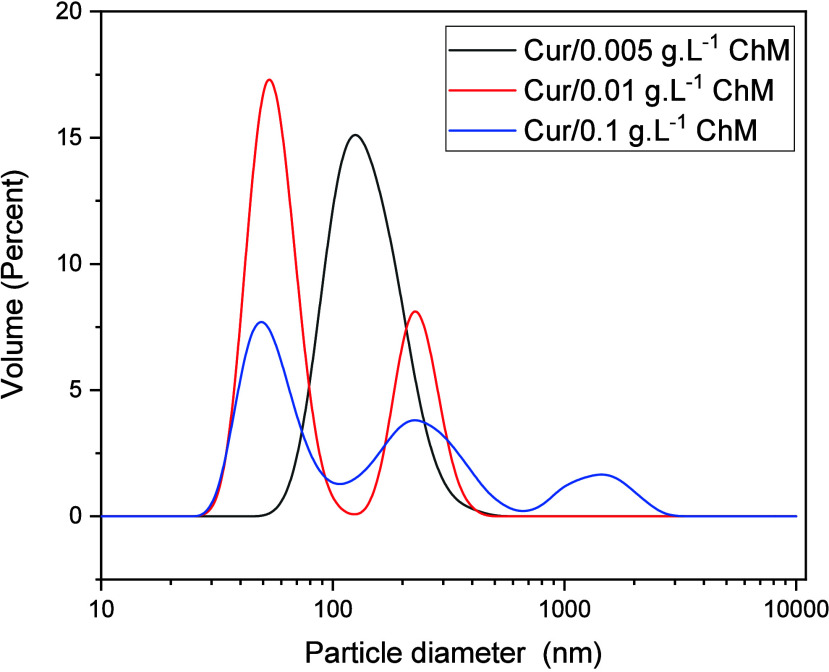
Particle size distribution in percentage of particle volume (DLS measurements) for modified chitosan with 2.5 × 10^–5^ mol. L^–1^ curcumin at three different ChM concentrations.

### 3.3 UV–Vis Absorption Spectra of Cur in Different Media as a Function of Time

UV–vis spectra of Cur solutions in different media as a function of time are presented in Figure 7. These spectra permit to follow Cur stability. Cur undergoes hydrolysis in water and produces *trans*-6-(4-hydroxy-3-methoxyphenyl)-2,4-dioxo-5-hexenal, ferulic methane, ferulic aldehyde, ferulic acid, and vanillin.^28^ Thus, as expected, the absorbance decreases with time for all samples due to Cur degradation.

**Figure 7:**
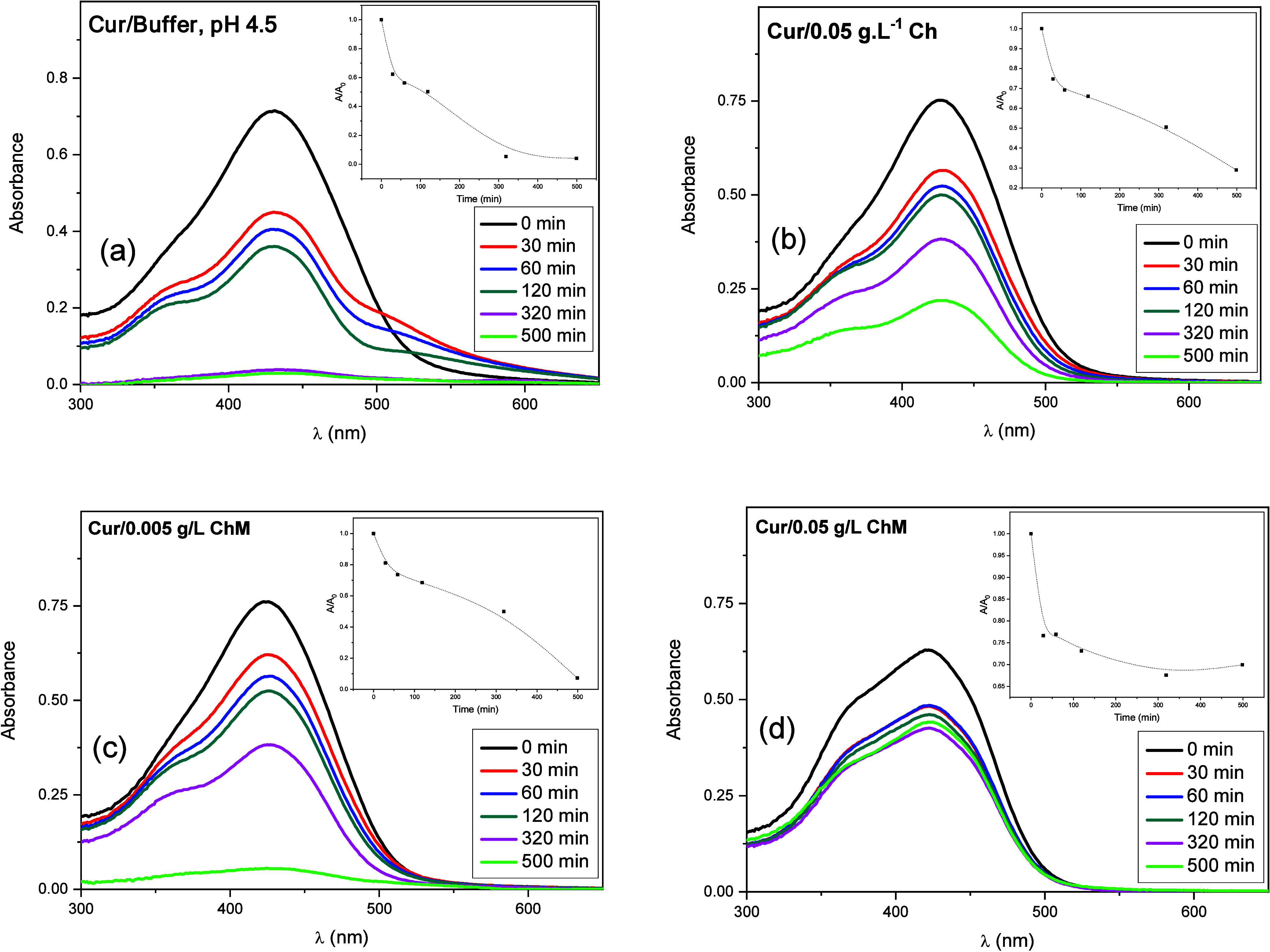
(a) UV–vis absorption spectra as a function of the time for curcumin (2.5 × 10^–5^ mol·L^–1^) in buffer, (b) Cur/chitosan and (c, d) Cur/ChM.

After 500 min, the percentage of Cur degradation was 96% in buffer and 71% in 0.05 g·L^–1^ Ch, 93% in 0.005 g·L^–1^ ChM, 60% in 0.01 g·L^–1^ ChM, and 30% in 0.05 g·L^–1^ ChM.

It is possible to observe that the Cur degradation percentage in 0.05 g·L^–1^ ChM (above CAC concentration) was lower when compared to the degradation of Cur in the other media.

Therefore, these results indicate that ChM above CAC, in its aggregated form, is able to protect the dye molecule from the hydrolysis that takes place in an aqueous medium.

Figure 8 presents the changes in absorbance at 420 nm as a function of time for the different media as a function of time.

**Figure 8:**
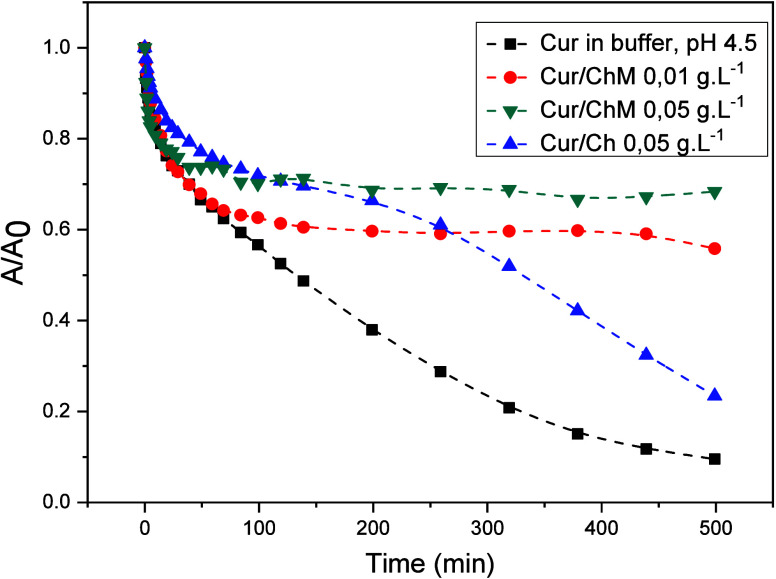
Changes in the absorbance at 420 nm as a function of time for curcumin in different media.

These curves reinforce that the presence of amphiphilic chitosan, when in concentrations above CAC, favors stabilization of the curcumin dye properties by means of a more hydrophobic environment provided by the inter- and intramolecular interactions.

Similar results were obtained in a previous work, in which it was reported that the Cur stability was increased in the presence of clays.^29^ However, in the present case, the stabilization took place in the presence of an organic, biocompatible system, allowing prediction of an enormous variety of applications in pharmaceutical and healthy areas.

## 4 Conclusions

The amphiphilic derivative of chitosan was successfully obtained by the synthetic route used. The hydrophilic DEAE and hydrophobic dodecyl groups were substituted at 49 and 17%, respectively.

The ChM aggregates at concentrations above 1.1 × 10^–2^ g·L^–1^, and hydrophobic environments are formed. The quantum yield value obtained for Cur in ChM was higher than that for Cur in the other media. Fluorescence micrographs revealed the fluorescent domains of Cur in the hydrophobic environments of the ChM. The percentage of Cur degradation in the ChM concentration above the CAC was lower compared to the Cur degradation in the other media. The stabilization of Cur was more significant in ChM at concentrations above the CAC, where the Cur molecule is concentrated in hydrophobic environments and protected from the aqueous medium. The results highlight the importance of modifying the polymer with the insertion of DD groups to improve the properties of the molecule.

These results also support future investigations of the Cur/ChM system and its application as a delivery strategy of Cur for pharmaceutical and medical applications.

## Supplementary Material



## Notes

Information on the electronic absorption and fluorescence spectra of cur/ChM in NaOH pH 9.0 (PDF)
