# Supplementary material for: Stability of Curcumin on Amphiphilic Chitosan
Source: ACS Omega. 2025 May 23;10(22):23082–8. doi: 10.1021/acsomega.5c01178 (PMC12163815; doi:10.1021/acsomega.5c01178)
Supplement: Supplementary file 1 [file ao5c01178_si_001.pdf]

Stability of Curcumin on Amphiphilic Chitosan

Alessandra L. Poli[a], Brenda G. Fanchiotti[a], Juliana S. Gabriel[a], Anderson M. Arandas[a,b] and Carla C. Schmitt*[a]

[a]Instituto de Química de São Carlos, Universidade de São Paulo, Caixa Postal 780, 13560-970 São Carlos SP, Brasil.

[b]Instituto Federal de Educação, Ciência e Tecnologia do Amapá – IFAP, Rodovia BR 210, Km 103, Zona Rural, 68997-000 Porto Grande AP, Brasil.

*Corresponding author: Carla C. Schmitt

E-mail address: carla@iqsc.usp.br

Tel. +55-16-3373-8685

SUPPORTING INFORMATION

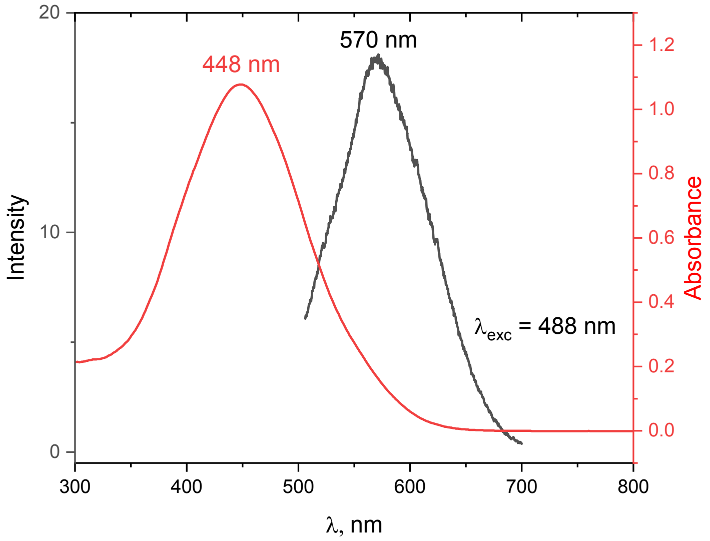

Figure S1. Electronic absorption and fluorescence spectra of Cur/ChM in NaOH pH 9.0.
